# Retraction and replacement of: Regulating community well-being through traditional mourning rituals: Insights from the Luhya People of Kenya

**DOI:** 10.1093/emph/eoaf013

**Published:** 2025-07-09

This is a retraction and replacement of: Stephen Asatsa, Sheina Lew-Levy, Stephen Ngaari Mbugua, Maria Ntaragwe, Wilkister Shanyisa, Elizabeth Gichimu, Jane Nambiri, Jonathan Omuchesi, Regulating community well-being through traditional mourning rituals: Insights from the Luhya People of Kenya, *Evolution, Medicine, and Public Health*, Volume 13, Issue 1, 2025, Pages 14–24, https://doi.org/10.1093/emph/eoaf001

Shortly after article publication, the Authors of this article alerted the Editorial Office to the presence of erroneous citations falsely generated by an AI language model used during their editing stage for grammatical corrections and enhancing the clarity of the article.

Given the nature of the changes and that no other results or conclusions were impacted by this error, the Authors are retracting the original version of the article and replacing it with a new version, which has been accepted by the Editors. The new version has been republished at https://doi.org/10.1093/emph/eoaf012.

